# Plant breeding is the food security basis in the Russian Federation

**DOI:** 10.18699/VJ21.039

**Published:** 2021-07

**Authors:** N.P. Goncharov, V.M. Kosolapov

**Affiliations:** Institute of Cytology and Genetics of the Siberian Branch of the Russian Academy of Sciences, Novosibirsk, Russia; Federal Williams Research Center of Forage Production and Agroecology, Lobnya, Moscow region, Russia

## Itnroduction

This issue of the Vavilov Journal of Genetics and Breeding is composed of reports of top Russian breeders delivered at the scientific session of the RAS Department of Agricultural Sciences “Scientific support of the efficient development of crop breeding and seed production in the Russian Federation” held in Moscow on December 7, 2020. This topic was chosen deliberately, as the food security concept in the Russian Federation determines the key directions and features of the modern development of Russian breeding. They involve the understanding and comprehensive analysis of breeding trends and the determination of prospects, particularly, in connection with import substitution1 and produce of next-generation cultivars.

The issue starts with the article by B.I. Sandukhadze et al. “Scientific breeding of winter bread wheat in the NonСhernozem zone of Russia: the history, methods and results”. It reviews the main steps and achievements of winter common (bred) wheat (Triticum aestivum L.) in the region throughout one century of scientific breeding. It shows that breeders’ efforts increased the yield of wheat cultivars to 14.0 t/ha, which is nearly ten times as high as in cultivars of early steps of scientific breeding in the central Non-Chernozem Area. Few residents of Moscow and Moscow region are aware of the “white spot” issue (a lot of rye was grown in the region in the early 20th century, as wheat production did not pay), successfully solved by prominent Russian breeder V.E. Pisarev by using early maturity cultivars from East Siberia. By now, cultivars produced by breeders of the FSC “Nemchinovka” have ensured the provision of the Non-Chernozem Area, a densely populated region of Russia, with locally produced food wheat grain and got the local population used to eating white bread. The Russian Federation is self-sufficient in producing not only wheat, barley, or oats but also rice (Gospadinova et al., 2016).

Area under grain legumes is second to cereals in Russia. They have accompanied cereals since the earliest steps of domestication on fields of ancient agriculturists. They diversified human diet and supplied domestic animals with high-protein fodder. The breeding of grain legumes is reviewed by V.T. Sinegovskaya “Scientific provision of an effective development of soybean breeding and seed production in the Russian Far East” and by V.I. Zotikov, S.D. Vilyunov “Present-day breeding of legumes and groat crops in Russia”. They note that soybean is becoming a crop of strategic importance for Russia and that groat crops constantly rank high in the diet of its inhabitants.

By import substitution we mean the substitution of imported goods and services for domestic ones. It implies the slowdown in the share of foreign manufacturers in the market and timely satisfaction of demand with domestic products.


The article by V.M. Lukomets et al. “Modern trends in breeding and genetic improvement of sunflower varieties and hybrids at VNIIMK” is dedicated to the breeding of the main oil plant in the Russian Federation. The Pustovoit AllRussian Research Institute of Oil Crops (VNIIMK), along with the Yuriev Plant Production Institute (Kharkiv, Ukraine) (Kirichenko et al., 2014) excels in the breeding of sunflower and other oil crops in the former Soviet Union.

The breeding of sugar beet, the main source of sugar in Europe, is considered by S.D. Karakotov et al. “Modern issues of sugar beet (Beta vulgaris L.) hybrid breeding”. The paper presents the results of monogerm varieties and successful application of molecular methods for testing the bred material of sugar beet.

The imbalance in fodder production that has existed in Russia for many years remains unresolved despite all the efforts of plant breeders. Even the considerable reduction in livestock in agricultural companies (agrofirms) and redistribution of large volumes of animal husbandry to private subsidiary farms (up to 50 % on the average) had no effect (Semenov, 2012). The article by V.M. Kosolapov et al. “Fundamentals for forage crop breeding and seed production in Russia” is dedicated just to this burning problem and potential ways to solve it. 

A series of three papers is dedicated to the breeding of fruit and small fruit crops, essential for balanced nutrition. It includes articles by E.A. Egorov “Grape breeding is a key link in the development of the grapes and wine-making industry”, I.M. Kulikov et al. “Scientific support of small fruit growing in Russia and prospects for its development”, and A.V. Ryndin et al. “Subtropical and flower crops breeding at the Subtropical Scientific Centre”. At present, import substitution draws attention to new (or, rather, well overlooked old) natural sources of vitamins and biologically active substances and to the breeding of domestic subtropical and flower plants

The progress in the breeding of medicinal and essential oil plants in Russia is considered by I.N. Korotkikh et al. “Breeding of medicinal and essential oil crops in VILAR: achievements and prospects”. This field became particularly important with regard to the sanctions, the ensuing shortage of herbal medicinal materials, and their poor quality, failing to meet the requirements of the present-day pharmaceutical industry.

Russian seed growers do not provide sufficient volumes of production of seeds of vegetable crops’ domestic varieties (Soldatenko et al., 2020). Modern breeding is based mainly on the gene pool at hand to involve older high-yielding varieties into breeding and improve them. The article by Yu.V. Fotev et al. “Genetic resources of vegetable crops: from breeding nontraditional crops to functional food” follows traditional VIR themes. It considers the introduction of untraditional crops in Russia in the context of the greatly requested area: their use in functional nutrition (Fotev et al., 2018).

The issue is concluded by N.P. Goncharov’s review “Scientific support to plant breeding and seed production in Siberia in the XXI century”. It emphasizes the importance of breeding activity in the development of Russian economy and the necessity for the preservation of still existing research institutions and units of abolished Breeding Centers in Siberia. In the contrary case, the consistency of breeding works in the region will disappear, and the unique breeding material created by generations of Russian scientists in research and breeding institutions will be lost beyond retrieval. The following problems are especially acute: Why cannot the federal and regional governments protect their intellectual property and preserve biodiversity of cultivated plants? What and who hampers? These issues, typical of Siberia, as well as the availability of skilled staffing concern other regions of Russia, too.

Several articles in the issue mention the necessity of the immediate solution of urgent tasks concerning the training of breeders in higher schools as a major component of food security in Russia. Nothing changes for centuries. In the end of the 19th century, A.S. Ermolov (1891) incriminated the backwardness of Russian agriculture to the absence of an agricultural education system and to the shocking ignorance of science among peasants.

It is pertinent to make a point about the publication policy of the Ministry of Education and Science and Presidium of the Russian Academy of Sciences. It is a sore point for not only agrarians but also the entire Russian academic community. For an unknown reason, the governmental strategy of import substitution does not apply to the scientific publishing activities. The principal journal “Selektsiya i Semenovodstvo” (Breeding and Seed Production) has ceased to be published. Specialized agricultural journals on particular crops or groups of crops demand a nation-specific policy. A.N. Engelhardt (1987) wrote that the agricultural science in its broad sense has pronounced “national” features: “There is no Russian, English, or German chemistry; there is only one chemistry for the entire world; but agronomy may be Russian, or English, or German, or else. <…> We should create Russian agricultural science of our own, and it can be created only by combined efforts of scientists and practicians, and there should be academically trained practicians in between” (p. 190).

It was repeatedly noted that different branches of Russian science need their own national platforms (journals) for communication and effective exchange of information. In particular, A.V. Yurevich and I.P. Tsapenko (2013) state that most Russian papers on socio-humanistic sciences are unfit for international journals not because of their flaws but because of the national specificity of their content. However, to bring studies in line with the themes of international journals means

to detach them from urgent Russian problems and to make the society think that the money of Russian taxpayers is spent in vain. Hence, the more patriotic is this or that branch of science and the more is it directed to the solution of domestic tasks, the less it fits into the international context. Even by the example of highly employable Vavilov’s studies we see that most of them are beyond the scope of interests of our Western colleagues, although they are conceptually important for the present-day global science. Neither Ministry of Education & Science, nor the current Presidium of the RAS see room for Russian journals in the world academic community. However sad it be, the task of any import substitution seems costly to Russian officials; therefore, the publication policy is the worst weakness of Russian science. We expressly indicate that the intellectual property of Russian scientists or Russia is not protected in publications in top Western journals, and it is often unaccessible for the scientific community in this country (Gorbunov-Posadov, 2020)

For many years, breeding in Russia has been distinguished by the widespread use of genetic knowledge. Breeding and genetics schools are held in Siberia on a regular basis since 1976 (Zilke, 2005). We can also mention the All-Siberia program ‘Diallel Analysis’ (Dragavtsev et al., 1984). Unfortunately, the gap between breeding and modern molecular biology is still unplugged.

Academician I.I.Artobolevskiy (1967) believed on reasonable grounds that the promotion of scientific achievements is a first-order duty of scientists. We try to find out why leading breeding schools in Russia insufficiently and reluctantly employ recent discoveries in molecular biology, biotechnology, and IT technologies. Presently, significant breeding achievements reached by using molecular methods exist in Russia (Bespalova et al., 2012; Pershina et al., 2020; among others). Promising studies opening up fresh opportunities for breeding are being conducted. In particular, the Institute of Cytology and Genetics (Novosibirsk) took part in the assembly of the wheat genome (IWGSC…, 2018). Here we are at the very beginning, since the information on the genome sequence from one accession is not sufficient to capture the whole spectrum of diversity in a gene pool responsible for phenotypic variation, plasticity, and environmental adaption. The de novo construction of a pan-genomes for cultivated plants is a mandatory step after the establishment of reference genome sequences for them. Obviously, it will be the key step in future breeding (Pronozin et al., 2021). The low sequencing depths even for wheat, a staple crop in Russia, still limit the broad use of pan-genomic analysis (Rasheed, Xia, 2019).

Several important problems concerning IT technologies in breeding were discussed in the previous issue of the Vavilov Journal of Genetics and Breeding, No. 1, 2021; so, we will not touch upon them. Nevertheless, the technological gap between the performance of genomic analysis and phenotypical description of plants is still large. In breeding a new variety, one should rest upon today’s perspectives and take into consideration both current requirements and remote prospects. Certainly, recent technological achievements in crop genomics generate new opportunities in the detection of genetic variations of traits important for breeding and permit one to create new-generation varieties. They come to the aid of breeders and allow fast, exact, and mass-scale description of plant phenotypes be it in the field or under laboratory conditions.

Functional genomics is a key to molecular breeding and basement for the development of diagnostic markers for gene introgression and molecular marker-assisted selection. Although the cloning of functional genes in crops was a slow process, few genes were cloned by conventional “positional cloning”. On the other hand, high-throughput PCR-based KASP (Kompetitive Allele-Specific PCR) markers (Rasheed et al., 2016) are helpful in the use of SNP arrays for high-density genotyping of wheat (Wang et al., 2014; Allen et al., 2017; Cui et al., 2017) and related species (Winfield et al., 2016), as well as in the annotation and introduction of functional genes. It is evident, though, that the role of money in the application of these methods to ordinary breeding is not the least, and they will not become widespread, inexpensive, and routine before long (Rasheed, Xia, 2019).

A characteristic feature of modern economy, including agriculture, is the predominance of novelties as a factor supporting competitiveness and economic advance in the long run. The question whether molecular biology can serve as a pioneering factor in plant breeding is open. Presently, prebreeding (assessment of the starting breeding material) is an application domain of up-to-date molecular methods. However, it is differently viewed by molecular biologists (Rasheed et al., 2016; Riaz et al., 2018), geneticists (Goncharov N.P. et al., 2020), and breeders (Bespalova et al., 2012). In contrast, their views on breeding itself (analysis of breeding material and selection process) are similar. Molecular methods are currently used to accelerate selection in the stabilization of breeding material (Adonina et al., 2021), its homozygotization (Pershina et al., 2020), and so on. The raise of promising breeding material can be accelerated by removing the germplasm of wild species from the progeny of introgression hybrids (Leonova et al., 2020); thereby, fragments of alien genetic material can be reduced in the genome of a species to breed (Adonina et al., 2021). This process is aimed at the reduction of adverse effects of concomitant genetic material transferred with the target genes. It has been demonstrated that molecular markers allow efficient selection for dwarfism (Kroupin et al., 2020; Sukhikh et al., 2021; etc.), early ripening (Kroupin et al., 2020), and many other traits. Meanwhile, the breeding for such important traits as crop productivity and quality of production is still conducted by conventional methods.

The assessment of the efficiency of plant genome editing by CRISPR/Cas technologies, operating with single functional genes, presents an acute and complicated problem (Chen et al., 2021). We do not know whether this one-gene manipulation is a breakthrough technology in breeding, which deals with hundreds of functional genes. Generally, there are no single genes whose replacement would result in sustainable progress. In addition, the CRISPR-edited plants have high somaclonal variability. Nevertheless, the tools and methods for plant transformation clearly alter phenotypes, being able to benefit from gene overexpression and other manipulations formerly inaccessible for breeders (Borisjuk et al., 2019).

Serious progress based on advanced technologies occurs in large seed producing companies, where breeders, geneticists, and molecular biologists work under the same roof. For instance, DuPond-Pioneer developed the Seed Production Technology concept, which successfully combines conventional hybridization with transgenic methods of raising malesterile (MS) lines, hybrid selection, and MS line support (Wu et al., 2016). The producing of nuclear MS lines by genome editing illustrates the applicability of this concept to wheat (Okada et al., 2019). Several more breakthrough technologies for hybrid creation are reviewed by Chen et al. (2021). The question remains open how soon this approach will become routine for breeding institutions.

Breeders’ work was scrutinized repeatedly. Nevertheless, we do not know how profoundly paradigm shifts in breeding (see the Figure) affect the speed of the breeding process and achievement of goals. It is doubtless that in recent decades traditional schemes involving hybridization and, to a much lesser extent, chemical and radiational mutagenesis contributed much to crop improvement. However, the globalization epoch necessitates the search for new groundbreaking methods. Many seemingly revolutionary methods came and went from the scientists’ toolkit and left an imprint only in breeding history records. Old-timers remember monosomic lines, which allowed the produce of varieties using the method of limited recombination and rapidly “repair” unique varieties. To tell the truth, the development of each from monosomic lines took 15–20 years. During this time, conventional breeders replaced the entire range several times and produced new remarkable varieties. It is natural that this approach did not provide a single commercial variety despite the huge scope of work (Worland, 1988). Protoplasts (Gleba, Sytnik, 1984), isoenzymes, and many others, looking modern in their days, did not change the breeding paradigm. It is worth mentioning that some breakthrough projects, such as domestication or green revolution (improvement of the range of wheat and rice varieties) were implemented by conventional breeding, and they were based on the choice of key traits regardless of the genetic and/or molecular mechanisms of their inheritance.

**Fig. 1.**
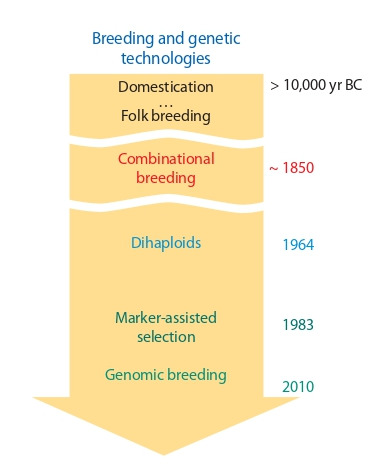
Milestones: the emergence of breakthrough technologies in plant breeding (after Kolchanov et al., 2017).

One of the targets of modern technologies is the acceleration of new variety breeding and introduction. For some reason, the powerful take it to mean the shortening of their producing time and make it the corner stone. It is a fringe concern, because the terms of variety submission to the Plant State Tasting System are insignificant in major breeding institutions, which produce series of new varieties massive. Present-day molecular Stakhanovites are nothing new. It is pertinent to recall the anecdote about the producing of cv. Lutescens 1163 common wheat by T.D. Lysenko et al. (1935) within 2.5 years by using know how: greenhouses and hybrids at hand instead of original accessions. It is sad that Stakhanovite methods of breeding are becoming nationwide again in the 21st century.

N.I. Vavilov likened a geneticist to a creator and stated that he “must act as an engineer; not only is he obliged to investigate his construction material, but he can and should construct new living species”2. The tasks are basically the same at present, it is the toolkit that has changed and expanded.

It is well known that genetics and breeding deal with heredity and variability and thus they interpenetrate. Breeding employs the laws of inheritance discovered by genetics, and genetics, in turn, obtains and generalizes data from breeding (Goncharov N.P., Goncharov P.L., 2018). While geneticists were seeking ways to overcome the abyss between genetics and breeding (Dragavtsev, 2005), molecular biology just revoked many of these problems (Moose, Mumm, 2008; Heffner et al., 2009; Abd-Elsalam, Lim, 2018; Ahmar et al., 2020). We have already mentioned that breeding received new tools. They provoked controversy as to whether they should be used extensively. Certainly, to know and master them is a must. However, business has an increasing share in the science of the 21st century. To conduct a modern study is a business operation. To obtain results is a business operation. To publish them in top-rated journals is a business operation. In the last case, to make a successful (in the eye of the Ministry of Education and Science) publication one should employ up-to-date expensive equipment, not necessary for the work itself. The organization of the breeding process is a business as well, since the development of crop production is increasingly considered only as the delivery of agricultural services. This situation reminds the notorious “arms race”, and we can win it by promoting our own rules.

The applied aspect of Russian science progressively increases (Rakin, 2020). Breeding in many European countries, including former COMECON members, is becoming private business under the pressure of multinational groups of agrochemical companies. The consequences are the list of top-priority research fields, the mainstream innovational practice of support by the Russian Science Foundation, the technological orientation of sections of the national Nauka project, and such. The national foundations of the Russian Federation do not imply considerable support of academic studies in breeding, solely the mastering and preservation of skills and technologies. Therefore, the search for alternative large sources of support for agricultural sciences, including 21st century breeding, is of paramount importance.

To conclude, we mention that breeding in the 21st century is directly associated with one of the global challenges, starvation. The 22nd session of the UN Food and Agriculture Organization of October 31, 1996, adopted the so-called Rome Declaration on World Food Security3, whose purpose was to halve the number of the starving on the Earth (800,000,000 as to 1996) by 2015. In fact, as reported by the German philanthropy organization Welthungerhilfe, the number of the starving had increased to one billion by 20204. With the current slow progress in increasing crop yields, 0.8 to 1.0 % annually, wheat, rye, and corn cannot be produced in quantities sufficient for the solution of the starvation problem

Thus, varieties play an essential role in improving the performance of global agricultural industry; therefore, breeders are seeking new sustainable, efficient, and cost-effective methods to produce new varieties. One of the major objectives of this issue of the Vavilov Journal of Genetics and Breeding is the thorough consideration of this task.
